# Promoting meaningful recovery with digital mental health care

**DOI:** 10.1017/S2045796020000165

**Published:** 2020-02-26

**Authors:** Elizabeth Carpenter-Song

**Affiliations:** Department of Anthropology, Dartmouth College, Hanover, NH, USA

**Keywords:** Community mental health, health service research, mental health, social anthropology

## Abstract

There is growing interest in digital mental health as well as accumulating evidence of the potential for technology-based tools to augment traditional mental health services and to potentially overcome barriers to access and use of mental health services. Our research group has examined how people with mental illnesses think about and make use of technology in their everyday lives as a means to provide insight into the emerging paradigm of digital mental health. This research has been guided by anthropological approaches that emphasise lived experience and underscore the complexity of psychiatric recovery. In this commentary I describe how an anthropological approach has motivated us to ask how digital technology can be leveraged to promote meaningful recovery for people with mental illnesses and to develop a new approach to the integration of technology-based tools for people with mental illnesses.

With over 300 000 mobile health apps currently available (IQVIA Institute for Human Data Science, 2017) and more than 400 million downloads of medical apps globally (Muoio, 2019), there is strong interest in using digital technology to support health. Within the area of mental health specifically, researchers have developed digital tools to address a range of needs, including supporting patient-provider communication regarding psychotropic medications (Deegan, 2010), providing knowledge and skills to help individuals cope with mental health symptoms (Ben-Zeev *et al*., 2014) and offering ongoing assessment of mental health conditions (Torous *et al*., 2015). In numerous studies, digital mental health tools have been found to be acceptable (Ben-Zeev *et al*., 2014) and some show promise in reducing psychiatric symptoms (Ben-Zeev *et al*., 2018). Emerging qualitative evidence highlights interest in using technology to support mental health among people with mental illness (Carpenter-Song *et al*., 2018). Among those who have used digital mental health apps, people were drawn to the ‘24/7’ availability of support through mobile technology (Carpenter-Song *et al*., 2020). As such, these tools are positioned to be useful ways to augment traditional mental health services and to potentially overcome barriers to access and use of mental health services.

Motivated to understand this emergent mental health treatment paradigm, our research group has examined how people with mental illnesses think about and make use of technology in their everyday lives. In this work, our approach has been guided by an anthropological orientation, which emphasises attention to lived experiences of mental illness (Jenkins, 2015) and ethnographic engagement with the culture of psychiatry (Luhrmann, 2000). Ahlin and Nichter (2015) noted that ‘few anthropologists have touched the topic [of e- and m-health]’ and ‘urged’ anthropologists to become involved. Indeed, the anthropological stance of questioning categories and interrogating assumptions (Lambert and McKevitt, 2002) provides a robust foundation for research focused on the phenomenon of digital mental health, as a means to temper the enthusiasm of the ‘medical imaginary’ (Good, 2001) with the complex lived realities of people with mental illness. Anthropological scholarship on psychiatric recovery engages with the ‘messy business of putting disrupted lived back together’ (Hopper, 2002) and underscores psychiatric recovery as involving personal ‘struggle’ with extraordinary experiences in the context of complex social realities that shape possibilities for improvement (Jenkins, 2015; Myers, 2015). Similarly, within mental health services research, scholars have long emphasised prioritising housing, employment and supportive social relations above narrowly symptom-focused efforts in promoting meaningful recovery (Drake and Whitley, 2014).

Placing a holistic understanding of recovery that extends beyond mitigation of psychiatric symptoms at the centre our work has motivated us to ask: How can digital technology be leveraged to promote meaningful recovery for people with mental illnesses? How, and to what extent, can digital tools address ‘what really matters’ (Kleinman, 2006)?

Our research has illuminated nuanced perspectives and experiences regarding technology among people with mental illnesses. We counter commonly espoused assumptions regarding the ubiquity of access to internet-enabled devices by documenting deep divides in ownership and quality of technologies owned by people with mental illnesses. For example, we found that clients in a rural community mental health centre commonly owned low-budget, pre-paid mobile phones with small data plans, which limited their ability to download apps. As a result, despite expressing interest in trying mental health apps, most participants were reluctant to use their limited data to download these products (Carpenter-Song *et al*., 2018). In addition, we found that despite the potential of technology to address mental health challenges, digital mental health tools were not in routine use among people with mental illnesses (Carpenter-Song *et al*., 2018) or in clinical settings (Noel *et al*., 2019*a*). Moreover, technology use was not commonly discussed between patients and mental healthcare providers (Noel *et al*., 2019*a*).

Building on these insights, we have developed a new approach designed to support people with mental illness and mental health providers to find and use digital technology to enhance recovery efforts. As we have described in more detail elsewhere (Noel *et al*., 2019*b*), the Technology Specialist is a new role within mental health care delivery. The Technology Specialist works in partnership with individuals with mental illness and mental health care providers to identify areas of recovery that could be addressed using technology. Unlike many research teams in the digital health space, we are not developing and evaluating our own technology-based tools. Instead, we are applying existing digital resources that we have evaluated for evidence, safety, privacy and usability (Torous *et al*., 2018). After a client selects a specific tool to use, the Technology Specialist provides ongoing support to address challenges with the technology as well as to assess if the tool is helping the individual to work toward their personal recovery goal.

This approach has encouraged us to cast our net wide in searching for tools that might be helpful. In most cases in our current field testing of the Technology Specialist intervention, participants wanted to use apps that would support them to engage in a broad range of activities, habits and behaviours that they viewed as part of their recovery processes such as meditation, exercise, cooking and smoking cessation, rather than more narrowly focused apps that address mental health symptoms. This resonates with our earlier research in which we found participants using technology to support their mental health in creative and unexpected ways such as engaging with positive daily affirmations on Instagram. In this way, our research is poised to reshape understandings of what ‘counts’ as a mental health tool toward a broader conceptualisation of tools that support the quotidian work of recovery (Jenkins and Carpenter-Song, 2005). The Technology Specialist may be a useful way to bridge people's interest in using technology to support mental health with readily available and vetted tools that are tailored to their needs and priorities. Moreover, by working in collaboration with both clients and mental health teams, this approach offers a way to introduce technology into clinical encounters.

In closing, I would like to reflect on some broader implications of our approach to digital mental health. First, our use of existing digital resources is responsive to the rapidly changing technological landscape. Our team uses real-time searches to find tools that may be of interest. Rather than viewing this process as eventuating in a static library of resources, we fully expect to iteratively refine and update over time. Second, through our immersion in existing digital resources, we have become aware of the overwhelming number of tools that currently exist as well as the redundancies across these tools. This has shaped our view that a movement toward open source rather than proprietary models within digital mental health would encourage creative collaboration and potentially reduce redundant efforts. A ‘digital mental health commons’ of this sort could promote new ideas, harness the speed of collaborative development and involve people with lived experience in the creation, evaluation and implementation of digital health tools. Finally, an anthropologically-informed perspective underscores the need for humility in recognising that technology-based tools are not panaceas. Close attention to the *struggle* (Jenkins, 2015) and *messy work* (Hopper, 2002) of living with mental illness reminds us that digital mental health tools need to be positioned within a broader context of professional and community supports that will cultivate possibilities for recovery. By placing meaningful recovery at the centre of digital mental health, we become attuned to both the promise and limitations of these new forms of mental health care.
